# Topology Optimization Design Method for Acoustic Imaging Array of Power Equipment

**DOI:** 10.3390/s24072032

**Published:** 2024-03-22

**Authors:** Jun Xiong, Xiaoming Zha, Xuekai Pei, Wenjun Zhou

**Affiliations:** School of Electrical Engineering and Automation, Wuhan University, Wuhan 430072, China; xiong19830000@163.com (J.X.); peixuekai@hotmail.com (X.P.); wjzhou@whu.edu.cn (W.Z.)

**Keywords:** acoustic imaging, topology design, power equipment, optimization algorithm

## Abstract

Acoustic imaging technology has the advantages of non-contact and intuitive positioning. It is suitable for the rapid positioning of defects such as the mechanical loosening, discharge, and DC bias of power equipment. However, the existing research lacks the optimization design of microphone array topology. The acoustic frequency domain characteristics of typical power equipment are elaborately sorted out. After that, the cut-off frequencies of acoustic imaging instruments are determined, to meet the needs of the full bandwidth test requirements. Through a simulation calculation, the circular array is demonstrated to be the optimal shape. And the design parameters affect the imaging performance of the array to varying degrees, indicating that it is difficult to obtain the optimal array topology by an exhaustive method. Aimed at the complex working conditions of power equipment, a topology optimization design method of an acoustic imaging array for power equipment is proposed, and the global optimal solution of microphone array topology is obtained. Compared with the original array, the imaging performance of the improved LF and HF array is promoted by 54% and 49%, respectively. Combined with the simulation analysis and laboratory test, it is verified that the improved array can not only accurately locate the single sound source but also accurately identify the main sound source from the interference of the contiguous sound source.

## 1. Introduction

There is no denying that it is of great significance for the safe and stable operation of a power system to find and deal with the latent defects of power equipment in time. The operating state information of power equipment is contained in vibration, sound, optical, electromagnetic, gaseous, temperature signals, etc. [1,2,3,4]. By effectively mining this rich operating state information, the health status of power equipment can be diagnosed, which enables us to capture and warn of latent defects as early as possible and avoid equipment failure or even accidents. Moreover, multiple signals can also be combined for diagnosis [5].

Among them, acoustic diagnosis technology has been extensively studied by experts and scholars due to its advantages of non-contact testing, no electromagnetic interference, and no impact on the power system. In fact, when mechanical defects [6,7,8], DC bias defects [9,10], or discharge defects [11,12] occur in power equipment, they make a sound that is obviously different from normal operation. Based on this, experts and scholars have conducted a lot of research on the diagnosis of abnormal acoustic signals, which focus on the preprocessing of sound signals [13,14,15], acoustic feature extraction [16,17], classifier design [18,19,20], etc. The above studies are based on a single sensor to determine whether there is an abnormal acoustic signal, but the specific location of the defect cannot be located. If a microphone array composed of multiple sensors is used to collect the same acoustic signal, it can locate defects intuitively and accurately based on the time difference between the acoustic waves reaching different sensors, which is called acoustic imaging.

There have been some studies and explorations of acoustic imaging technology. In Ref. [21], three-microphone sensor arrays are used, and two methods for estimating the angle of arrival (AOA) and the time difference of arrival (TDOA) are proposed to achieve the high-precision localization of sound sources. In Ref. [22], a four-microphone sensor array is composed, and then an accurate position calculation method based on pure geometric phase transformation is proposed, which has higher positioning speed and accuracy. In Ref. [23], a spherical sensor array is designed to achieve full bandwidth positioning function. However, the above array is not developed for the acoustic imaging of power equipment, in which only a small number of sensors are used. The topology of the array has not been logically meticulously designed. In Ref. [24], a reverberation robust feature extraction method for sound source localization based on sound intensity (SI) estimation is proposed, which realizes microphone array miniaturization. In Refs. [25,26], two novel positioning methods are proposed to improve the calculation speed of a high-resolution and large-scale matrix, which greatly reduces the calculation cost without losing accuracy. In Refs. [27,28], a non-synchronous array measurement is elaborated to break through the beamforming barrier of acoustic imaging in the low-frequency domain. The above research has focused on improving imaging algorithms to enhance positioning speed, accuracy, and economy. However, they ignore that changing the microphone array topology is also expected to further improve the positioning performance. In fact, an unreasonable topology design can lead to poor imaging effects and high costs. If an exhaustive method is used to find the optimal topology, it would consume a lot of simulation computing resources and time, and it is difficult to obtain the optimal solution. Therefore, it is urgent to explore how to reasonably establish a mathematical model of topology optimization and propose a corresponding optimization method.

The remainder of this paper is organized as follows. Section 2 briefly introduces some basic principles and concepts of acoustic imaging technology. In Section 3, the influence of different design parameters on microphone array performance is explored. A topology optimization design method of a microphone array for power equipment is proposed in Section 4. Brief conclusions are drawn in Section 5.

## 2. Basic Principles of Acoustic Imaging Technology

### 2.1. Process and System Composition of Acoustic Imaging

**Figure 1:**
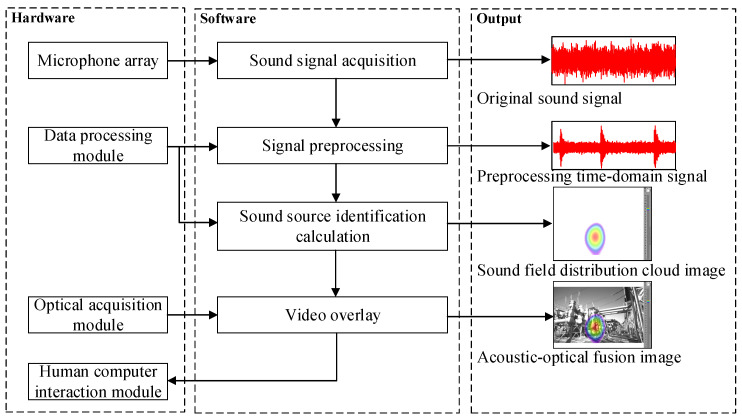
Schematic diagram of acoustic imaging system.

Sound source localization is realized through sound signal acquisition, sound signal preprocessing, sound source identification calculation, and video overlay, as shown in Figure 1. The specific steps are as follows.

1. The microphone array collects the original time domain sound signal.
2. The time domain signal is preprocessed, including two types of operations. They are noise reduction and amplitude enhancement.
3. The sound source distribution information on the sound source-focusing plane is calculated in real-time, so that the sound field distribution cloud images are formed.
4. The real-time optical images can be obtained through the optical acquisition module.
5. The sound field distribution cloud image is transparently superimposed on the optical image, forming the acoustic–optical fusion image.
6. The sound source spatial position can be very intuitive to see from the fusion image which parts of the power equipment show the most obvious acoustic characteristics. It should be noted that through the fusion image, not only can a single sound source be clearly identified but it is also expected to accurately identify multiple sound sources.

### 2.2. Sound Source Identification Algorithm

**Figure 2:**
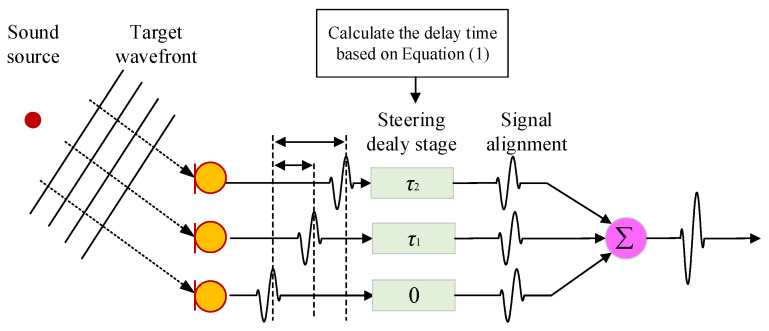
Schematic diagram of overall process of DSB algorithm.

Sound source identification algorithms can be divided into two categories: adaptive beamforming and spatial spectrum estimation. Among them, the Delay Sum Beamforming (DSB) algorithm is widely used as a classic and mature adaptive beamforming algorithm due to its advantages of its simple principle and high computational efficiency. The overall process of the DSB algorithm is shown in Figure 2 [29,30]. Its working principle is as follows.

**Figure 3:**
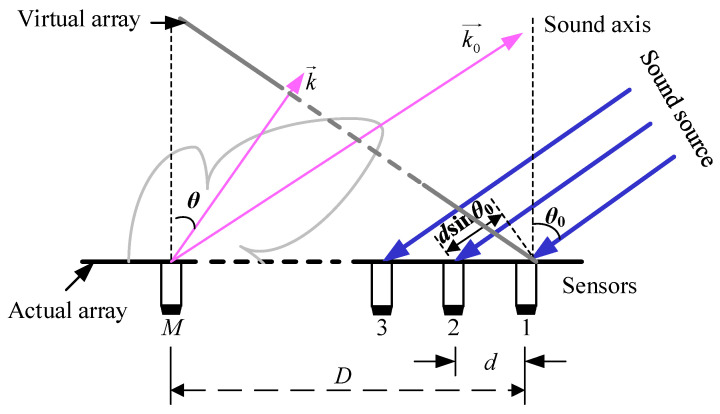
Schematic diagram of linear array.

Assuming in a linear array, there are *M* sensors arranged at equal distances, as shown in Figure 3. The distance between two adjacent sensors is equal and represented by *d*. The angle between the array sound axis and the direction of sound waves is *θ*_0_. The specific description of the DSB algorithm process is as follows.

Step 1: According to the given scanning angle *θ*, the time delay between a sensor and the reference sensor is calculated. In Figure 3, sensor 1 is set as the reference sensor, and the time delay of each sensor *τ_m_* can be calculated according to Equation (1).

(1) $$\tau_{m}=\frac{(m-1)d\sin\theta }{c}$$

where *m* is the serial number of the sensor, and *c* is the speed of sound propagation.

Step 2: By compensating for the time delay of the signal received by each sensor, the actual array can be rotated to the virtual array.

Step 3: The signal received by each sensor is weighted, to control the side lobe.

Step 4: The output of the DSB algorithm is obtained by adding the signals of all sensors together:

(2) $$B(\theta ,t)=\sum_{m=1}^{M}w_{m}p_{m}\left(t-\tau_{m}\right)$$

where *w_m_* is the *m*th weighting coefficient; *p_m_* is the sound pressure signal received by the *m*th sensor.

According to the DSB algorithm, the relationship between the output *B*(*θ*,*t*) and the array scanning angle *θ* can be obtained. The output changes with *θ* changes from 0° to 360°. At *θ* = *θ*_0_, the output reaches the maximum, and this angle is the direction of the sound source.

### 2.3. Acoustic Characteristics of Power Equipment

There are many previous studies on voiceprint detection for power equipment such as gas insulated switchgear (GIS), power transformer, reactor, etc. The results indicate that all kinds of power equipment show typical acoustic characteristics.

#### 2.3.1. Acoustic Frequency Domain Characteristic Parameters

1. Dominant frequency. It refers to the frequency with the highest amplitude in the spectrum.
2. Proportion of odd harmonics (POH). It refers to the proportion of 50 Hz odd-fold frequency in total power, as shown in Equation (3).

(3) $$P_{\mathrm{odd}}=\sum_{i=1}^{\infty }p_{h}(100i-50)/p_{\mathrm{total}}\times 100\%$$

where *p*_h_(*f*) is the power at frequency *f*, and *p*_total_ is the total power.

3. Proportion of even harmonics (PEH). It refers to the proportion of 50 Hz even-fold frequency in total power, as shown in Equation (4).

(4) $$P_{\mathrm{even}}=\sum_{i=1}^{\infty }p_{h}(100i)/p_{\mathrm{total}}\times 100\%$$

4. Proportion of accumulated energy (PAE). It refers to the proportion of acoustic cumulative power within the frequency range of *f*_L_ to *f*_H_, as shown in Equation (5).

(5) $$E=\int_{f_{L}}^{f_{H}}p(f)dt/p_{\mathrm{total}}\times 100\%$$

#### 2.3.2. Acoustic Frequency Domain Characteristics

Abnormal operating conditions such as loose bolts, partial discharge, and breakdown discharge may occur in GIS. Abnormal operating conditions such as over excitation, DC magnetic bias, and iron-core multi-point earthing may occur in the power transformer or reactor. Table 1 summarizes the frequency domain characteristics of different power equipment. As shown in Table 1, under different operating conditions of power equipment, the dominant frequency varies from 100 Hz to 10 kHz or even higher, showing a wide frequency distribution characteristic.

## 3. Influence of Design Parameters on Performance of Microphone Array

The microphone array is composed of multiple acoustic sensors arranged according to certain rules. In this section, the influence of the design parameters, such as the array shape, number of sensors, number of rings, and rotation angle of rings on array performance is discussed for a single sound source. This provides theoretical support for subsequent optimization analysis.

### 3.1. Performance Evaluation Indexes of Microphone Array

The common performance evaluation indexes of the acoustic array include the directivity function, main lobe width (MLW), maximum side lobe level (MSL), cut-off frequency, etc.

1. Directivity function

The directivity function $W\left(\vec{k}-\vec{k_{0}}\right)$ indicates the ratio of the output of the array in a certain focusing direction $\vec{k}$ to the main pointing direction $\vec{k_{0}}$:

(6) $$W\left(\vec{k}-\vec{k_{0}}\right)=\frac{1}{M}\left|\sum_{m-1}^{M}w_{m}e^{j\left(\vec{k}-\vec{k_{0}}\right)}•\vec{r_{m}}\right|$$

where $\vec{k}$ is the wave vector in any direction. $\vec{k_{0}}$ is the wave vector in the focusing direction. *M* represents the number of sensors. $w_{m}$ is the *m*th sensor weighting coefficient. $\vec{r_{m}}$ is the *m*th sensor position vector.

**Figure 4:**
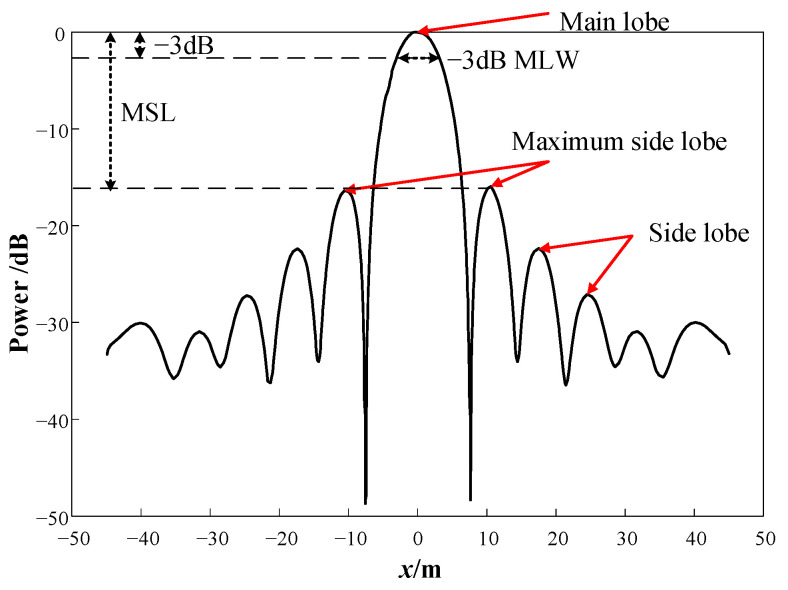
Beam pattern of beamforming algorithm after normalization processing.

The beam pattern can be obtained from the directionality function, as shown in Figure 4. The mapping relationship between the beam function and the directionality pattern is described as follows.

The location *x* is shown in Equation (7).

(7) $$x(\zeta )=2d_{\min}\tan\left(\frac{\zeta }{2}\right)$$

where ξ is the angle between $\vec{k}$ and $\vec{k_{0}}$; *d*_min_ is the minimum distance between sensors.

The power value *P* is shown in Equation (8).

(8) P=−10lg(W)

2. −3 dB MLW

The main lobe refers to the highest peak in the beam pattern. The −3 dB MLW refers to the width of the main lobe when the power is half of the maximum power. The narrower the −3 dB MLW is, the better the imaging performance is.

3. MSL

The MSL refers to the ratio of the maximum side lobe to the main lobe. Since the beam pattern in Figure 4 has been normalized and transformed into a logarithmic form, the MSL can also be understood as the difference between the maximum side lobe and the main lobe. Therefore, the smaller the MSL is, the better the side lobe suppression effect is, and the main lobe is more prominent.

4. Cut-off frequency

According to Nyquist’s law, the array aperture *D* should not be less than twice the maximum wavelength λ_max_ corresponding to the lower cut-off frequency *f*_min_. It can be expressed in Equation (9).

(9) $$f_{\min}=\frac{2c}{D}$$

The upper cut-off frequency *f*_max_ is obtained by the spatial sampling law.

(10) $$f_{\max}=\frac{c}{d(1+\sin\varphi )}$$

where *φ* is the array opening angle.

The −3 dB MLW and the MSL are usually used to describe the sound source localization effect. Therefore, these indexes are chosen in this paper.

### 3.2. Preliminary Design of Microphone Array

Considering the wide frequency bandwidth distribution of sound signals under different operating conditions of power equipment, the microphone array in a single frequency bandwidth is difficult to meet the full bandwidth test requirements. This paper will design two sets of instruments, which are low-frequency (LF) and high-frequency (HF) instruments.

1. LF instrument

According to Equation (9), the reduction in the lower cut-off frequency will increase the array size, thus increasing the carrying difficulty and manufacturing cost. At the same time, considering the safe distance of the live power equipment, the array with a diameter of 1.7 m is selected. The corresponding lower cut-off frequency is 400 Hz. Frequency components of 5 kHz or above occur in the spectrum as the discharge arises. In order to ensure full frequency coverage, the upper cut-off frequency is fixed at 7 kHz.

2. HF instrument

If partial discharge or breakdown discharge occurs, a large number of signal characteristic signals will be observed at 5 kHz and above. Therefore, 5 kHz is selected as the lower cut-off frequency of the HF instrument, and the corresponding array diameter is 0.136 m. At the same time, considering that there are few characteristic signals above 20 kHz, the upper cut-off frequency is selected at 20 kHz.

### 3.3. Influence of Array Shape

**Figure 5:**
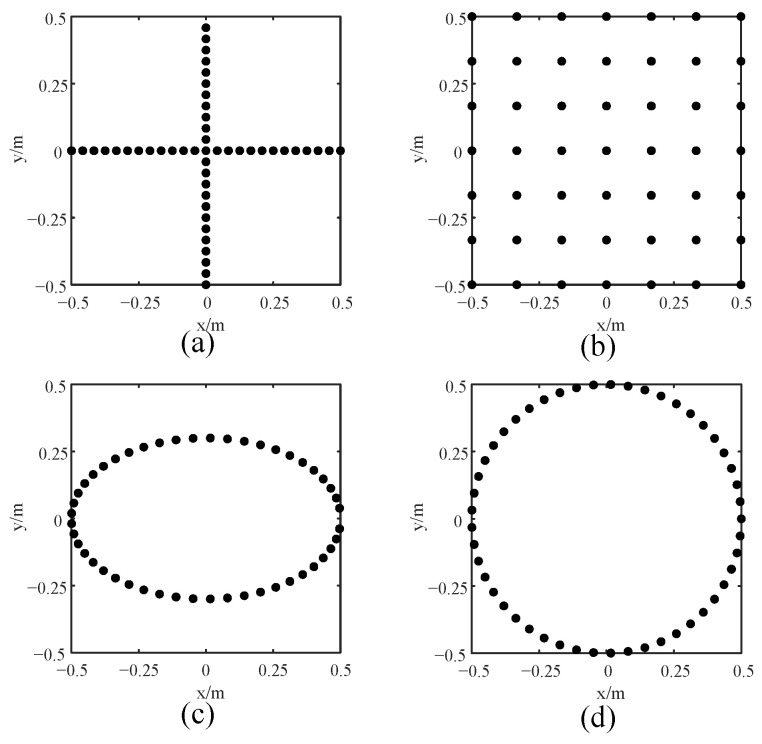
Array shape diagram: (**a**) crossed array, (**b**) rectangular array, (**c**) elliptic array and (**d**) circular array.

**Figure 6:**
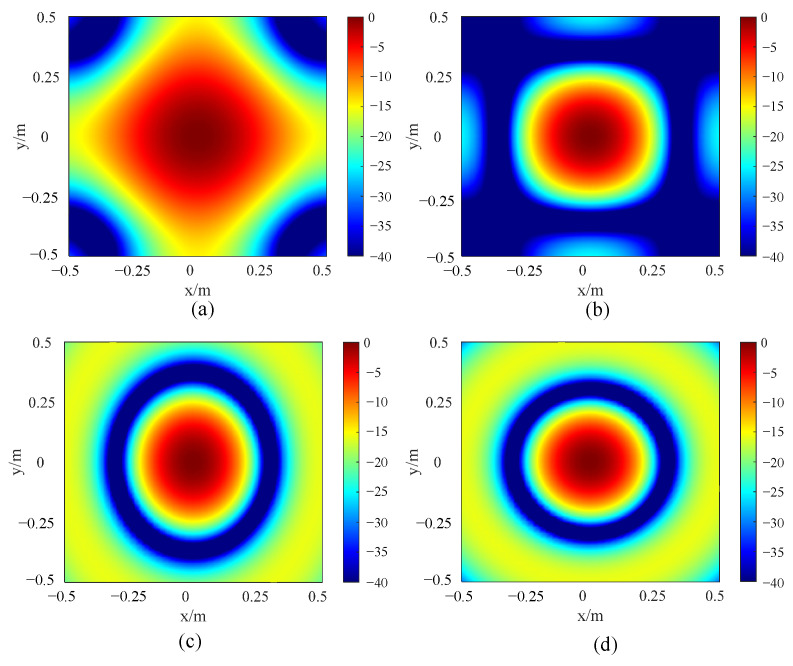
The 2D response images of arrays with different shapes: (**a**) crossed array, (**b**) rectangular array, (**c**) elliptic array and (**d**) circular array.

The commonly used arrays are crossed, rectangular, elliptic. and circular, as shown in Figure 5. A single sound source is set at 1 m away from the microphone array. The size of the array is limited to 1 m × 1 m, and the number of sensors is fixed at 49. Based on the MATLAB simulation platform, the simulation analysis of the imaging effect of the different arrays is carried out. The sound source is set at a distance of 1 m from the center of the array, and its acoustic frequency is set to 1 kHz. Then, 2D response images are obtained, as shown in Figure 6.

Table 2 summarizes the −3 dB MLW and MSL. It can be seen that both the −3 dB MLW and the MSL of the circular array are the smallest. Actually, the smaller the two evaluation indexes are, the better the imaging effect is. It can also be seen intuitively from Figure 6 that the main lobe in the circular array response image is the most focused, and the side lobe suppression effect is the best. From two perspectives of quantitative analysis and response image analysis, the circular array is selected in the following text for further performance improvement.

### 3.4. Influence of Number of Sensors

**Figure 7:**
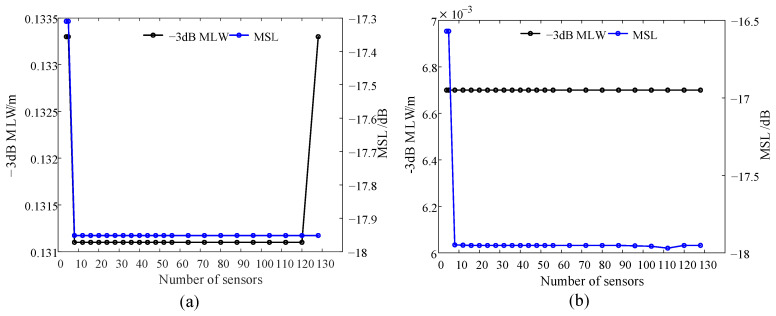
Influence of number of sensors on evaluation indexes: (**a**) lower cut-off frequency and (**b**) upper cut-off frequency.

Taking the LF instrument as an example, the influence of the number of sensors on the array performance is studied by simulation analysis. Sensors are evenly distributed on a ring. Figure 7 shows the analysis results.

At 400 Hz, the −3 dB MLW and MSL almost do not change with the number of sensors when the number exceeds eight. The main reason is that the −3 dB MLW is only related to the array diameter and has little to do with the number of sensors. It should be noted that the change trend of 3 dB MLW at 7 kHz is independent of the number of sensors and that the change trend of the MSL is similar to that at 400 Hz.

The MSL reflects the ability of the microphone array to suppress the side lobes. The reduction in the MSL may also improve the performance of the microphone array in terms of anti-jamming, particularly in complex environments such as substations. In these scenarios, there are a large number of adjacent interference sound sources, such as noise interference from non-detection target power equipment during normal operation. If no means are taken to suppress the side lobe, the weak sound source may be submerged by the adjacent strong background noises. Furthermore, excessive side lobes are also responsible for false targets and virtual images. Consequently, the subsequent research mainly focuses on the anti-interference performance of the microphone array, which is quantified by the size of the MSL.

### 3.5. Influence of Number of Rings

**Figure 8:**
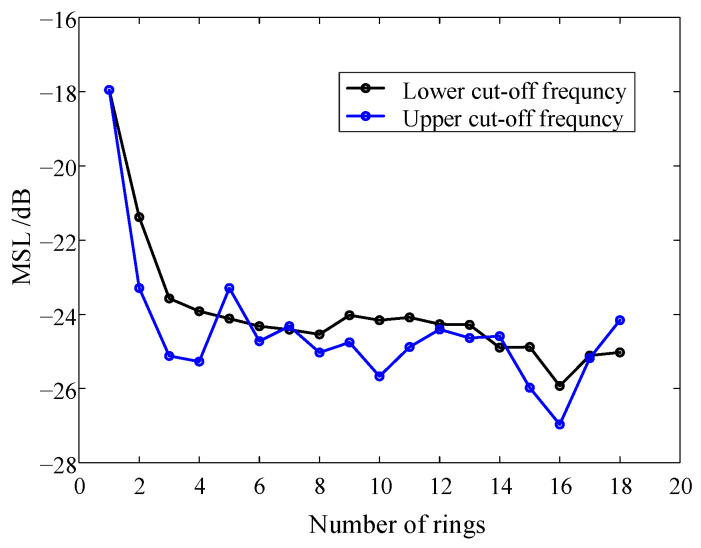
The influence of the number of rings on the MSL.

The sensors on multiple rings are radial. That is, the rotation angle of each two rings is 0. The sensors are evenly distributed on multiple rings. The sensor interval on a ring *d*_s_ is equal, and the distance between each two adjacent rings *d*_r_ is equal. Taking the LF instrument as an example, the number of sensors is fixed at 112, and the number of rings varies from 1 to 18. On this basis, the MSL at the cut-off frequencies is calculated, respectively. It should be noted that the lower the characteristic frequency of the defect is, the harder it is to locate the defect. So, the positioning performance of the instrument at the lower cut-off frequency is the most valued and most representative. In addition, in order to verify the positioning effect of the HF defect, the upper limit frequency test group is added. The results are shown in Figure 8.

**Figure 9:**
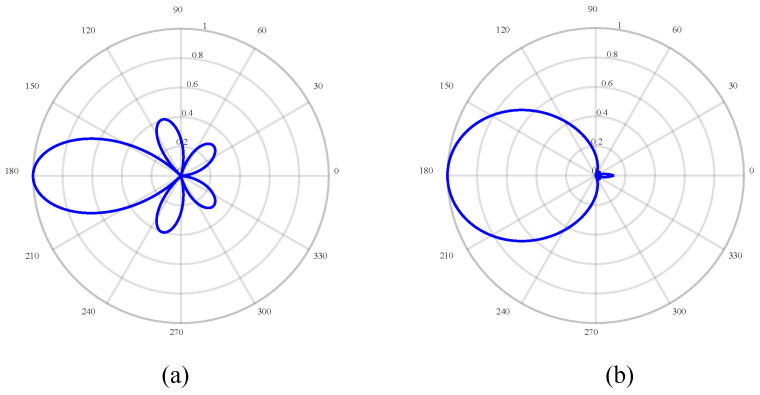
Comparison of directivity functions under different number of rings: (**a**) number of rings is 1 and (**b**) number of rings is 16.

In Figure 8, at the lower cut-off frequency, the MSL shows a downward trend on the whole, mainly for the following reasons. On the one hand, as the number of rings increases, *d*_s_ increases and *d*_r_ decreases. The increase in *d*_s_ leads to the increase in the MSL. In contrast, as *d*_r_ decreases, the MSL decreases. It is dependent upon which of the two contradictory parameters has a greater influence on the trend of the MSL. The MSL is smallest when the number of rings is 16. The directivity functions with the number of rings of 1 and 16 are compared, as shown in Figure 9. As it can be observed, when the number of rings is 16, the side lobe is very small, and the anti-interference ability is the strongest. When the frequency is 7 kHz, the MSL change rule is similar to that of 400 Hz.

### 3.6. Influence of Rotation Angle of Ring

**Figure 10:**
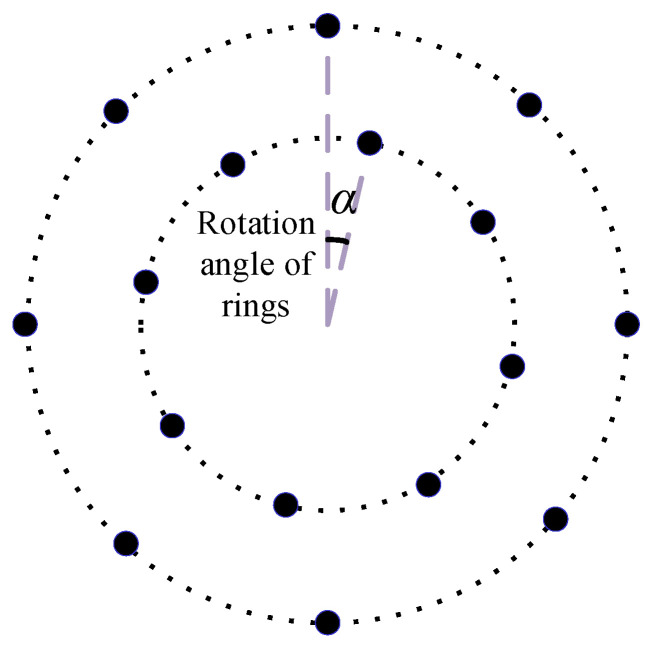
Schematic diagram of rotation angle.

**Figure 11:**
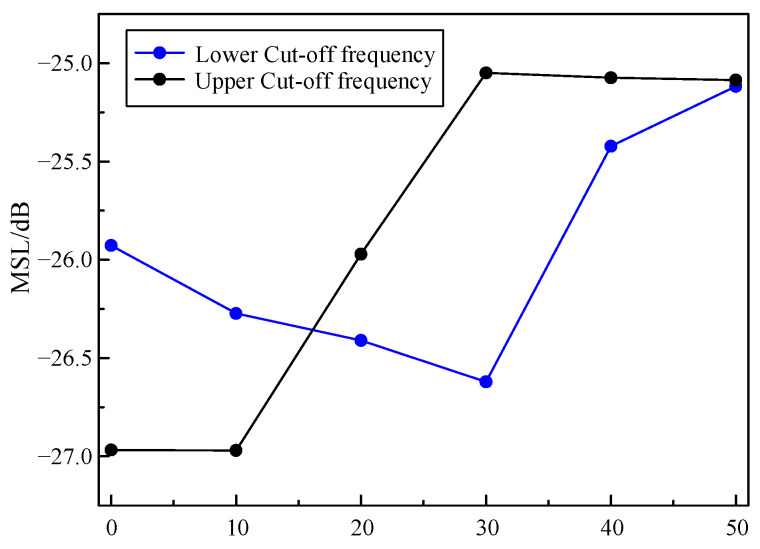
Influence of rotation angle on array performance.

For two adjacent rings, the anticlockwise rotation angle of the outer ring relative to the inner ring is the rotation angle α, as shown in Figure 10. Taking the LF instrument as an example, the influence of α on the array performance is studied. The number of rings is fixed at 16, and the number of sensors is set to 112. The rotation angle α ranges from 0° to 50°. The MSL is obtained as shown in Figure 11.

Based on Figure 11, it is evident that at 400 Hz, the MSL shows a trend of decreasing first, followed by an increase, with a minimum value at α = 30°. This indicates that an appropriate rotation angle can improve the anti-interference ability of the array, while an excessive rotation angle will degrade the performance of the array.

At 7 kHz, as the rotation angle increases, the MSL begins to increase and then remains stable, indicating that the excessive rotation angle has a negative impact on array performance. The simulation results prove that rotating at a certain angle to obtain better array performance at the lower cut-off frequency is at the expense of array performance at the upper cut-off frequency.

## 4. Optimization Design of Microphone Array

As mentioned above, it is difficult to obtain the optimal array design parameters by a simple exhaustive method. Therefore, it is necessary to propose an optimization design method of array topology for the complex noises of power equipment.

### 4.1. Mathematical Modeling of Optimization Problem

As noises in substations are complicated, the lower the MSL is, the greater the ability of the array to suppress spatial confusion will be. Therefore, the optimization problem can be described as follows:

(11) $$\left\{\begin{array}{l} \min\left\{f(m,n,r_{i},\theta_{j})\right\}\\ s.t.\left\{\begin{array}{l} m\in [m_{\min},m_{\max}]\\ n\in [n_{\min},n_{\max}]\\ r_{i}\in [r_{\min},r_{\max}]\\ \theta_{j}\in [\theta_{\min},\theta_{\max}]\\ m=k\times n,k=1,2,3,... \end{array}\right. \end{array}\right.$$

where *f*(*m*, *n*, *r_i_*, *θ_j_*) represents the MSL function. *m* represents the number of sensors. *n* is the number of rings. *r_i_* is the radius of each ring. *θ_j_* is the rotation angle of each ring. *m*_min_ and *m*_max_ are the minimum and maximum values of the number of sensors. *n*_min_ and *n*_max_ are the minimum and maximum values of the number of rings. *r*_min_ and *r*_max_ are the maximum and minimum radius of each ring. *θ*_min_ and *θ*_max_ are the minimum and maximum values of the rotation angle of each ring. *i* = 1, 2, …, *n*. *j* = 1, 2, …, *n* − 1.

### 4.2. Optimization Algorithm

**Figure 12:**
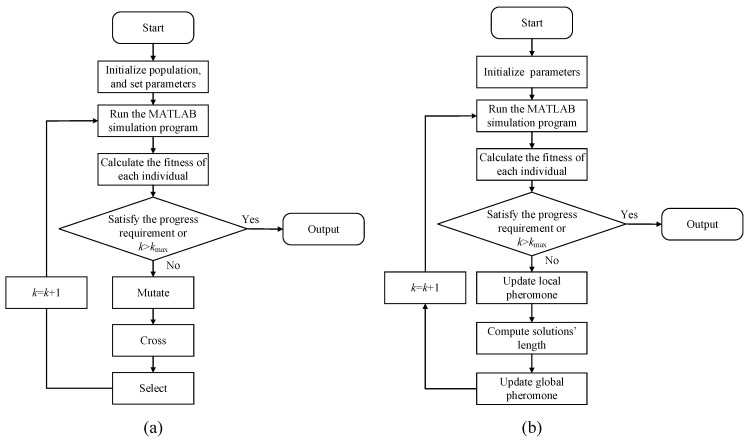
Algorithm flow: (**a**) DEA and (**b**) ACA.

The differential evolution algorithm (DEA) has the advantages of good convergence, simple principle, and strong robustness. It is suitable for solving nonlinear problems. As a result, the DEA is adopted to solve the above problem. The algorithm flow is shown in Figure 12a, including initialization, fitness calculation, mutation, crossover, and selection.

### 4.3. Optimization Results

#### 4.3.1. LF Instrument

As mentioned above, the outermost ring diameter of the LF instrument is set to 1.7 m, and other boundary conditions are listed in Table 3.

**Figure 13:**
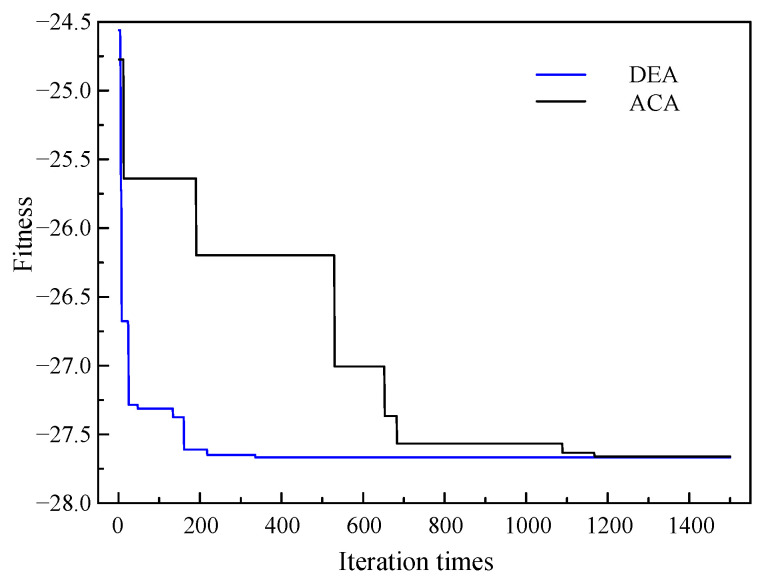
Convergence characteristics of fitness function of LF instrument.

The optimization results are shown in Table 4. The convergence characteristics of the fitness function is shown in Figure 13. As can be seen, when the iteration times exceed 336, the fitness approaches the minimum and tends to remain constant. It appears that the objective function may have reached its global optimum solution. In order to verify that the objective function does not fall into the local optimum solution, the ant colony algorithm (ACA) is used to solve the same model. The algorithm flow is shown in Figure 12b. It can be seen that when the iteration times exceed 1169, the objective function reaches the minimum value, which is the same as the DEA calculation result, indicating that the result is the global optimal solution.

**Figure 14:**
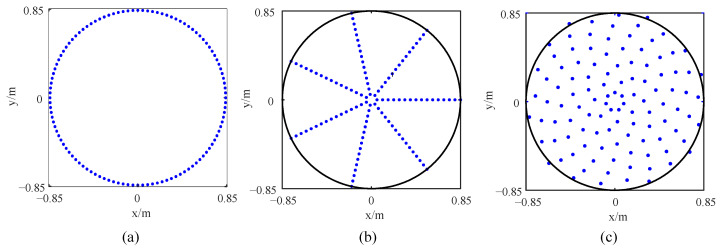
Topology of LF arrays: (**a**) original array, (**b**) quasi improved array and (**c**) improved array.

**Figure 15:**
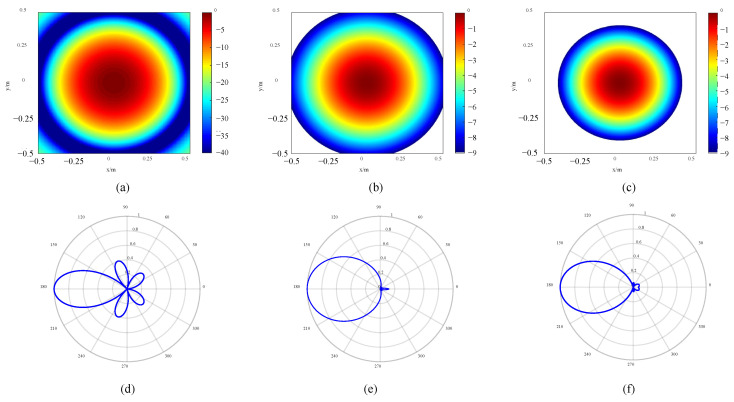
Response of LF arrays: (**a**) 2D image of original array, (**b**) 2D image of quasi improved array, (**c**) 2D image of improved array, (**d**) directivity of original array, (**e**) directivity of quasi improved array and (**f**) directivity of improved array.

To verify the performance optimization effect of the improved array, the original array is designed, which is consistent with the number of sensors of the improved array. All sensors in the original array are equidistantly distributed on a ring, as shown in Figure 14a. The sensors in the quasi-improved array are evenly distributed on multiple rings, and the number of rings and number of sensors are consistent with the improved array. At the same time, the distance between each two adjacent rings *d*_r_ in the quasi-improved array is equal, and the rotation angles *θ_j_* are all set to be zero, as shown in Figure 14b. According to Table 4, the topology of the improved array can be drawn, as shown in Figure 14c. In particular, the quasi-improved array is a special array obtained by optimizing the number of sensors and the number of rings. Actually, if these two parameters are uncertain, it is difficult to obtain the quasi-improved array by the exhaustive simulation analysis and comparison one by one. The response of the arrays is shown in Figure 15. The comparison of the MSL can be found in Table 5.

It can be seen from Table 5 that compared with the original array, the quasi-improved array evenly distributes 112 sensors on 16 rings, so the MSL is increased by 44.46%, which shows that the optimization method proposed in this paper can guide the topology design and avoid blind design. In addition, rotating by a certain angle and adjusting the interval between the rings can further improve the positioning effect. Compared with the original array, the effect is improved by 54.15%, which is also an effect that cannot be achieved by the exhaustive method.

#### 4.3.2. HF Instrument

**Figure 16:**
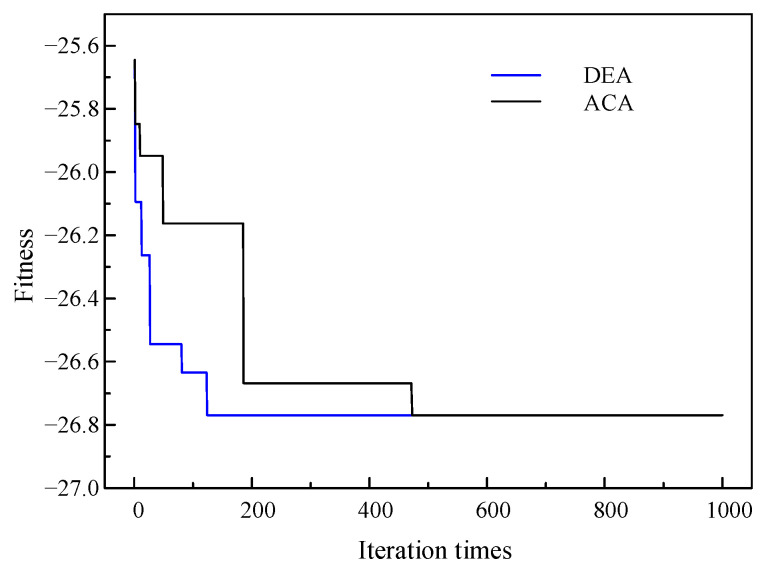
Convergence characteristics of fitness function of HF instrument.

Based on the boundary conditions shown in Table 6, the optimal microphone array topology can be obtained through the optimization algorithm, as shown in Table 7. Figure 16 shows the convergence characteristics of the fitness function. It can be seen that the curve change rule is similar to the LF instrument. The DEA and the ACA both produce the same result, indicating that this result is the global optimal solution.

**Figure 17:**
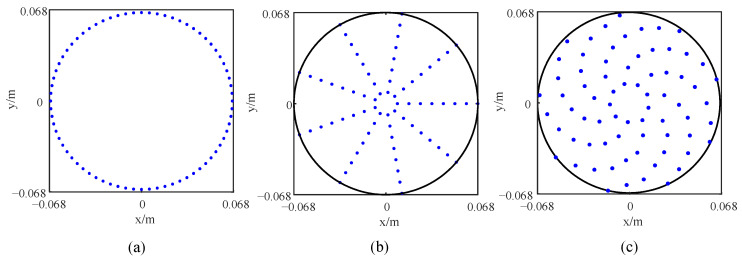
Topology of HF arrays: (**a**) original array, (**b**) quasi improved array and (**c**) improved array.

**Figure 18:**
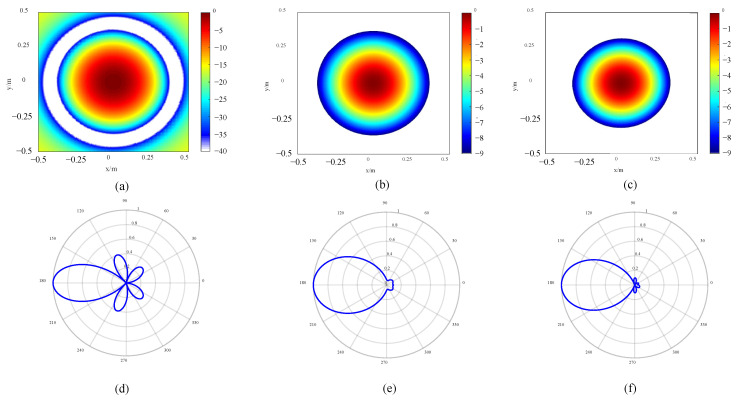
Response of HF arrays: (**a**) 2D image of original array, (**b**) 2D image of quasi improved array, (**c**) 2D image of improved array, (**d**) directivity of original array, (**e**) directivity of quasi improved array and (**f**) directivity of improved array.

The topology of the HF original and improved arrays is shown in Figure 17. The response of the arrays is presented in Figure 18. The MSL is compared in Table 8. It can be found that compared with the original array, the quasi-improved array and the improved array enhance the performance by 42.74% and 48.97%, respectively. From Figure 18, it can also be seen intuitively that the spot size of the quasi-improved array is significantly reduced, and the side lobe is obviously suppressed. On this basis, the spot size of the improved array is further diminished.

To sum up, through the optimization method proposed in this paper, whether it is to obtain the quasi-improved array or the improved array, a very significant performance improvement can be obtained, which verifies the effectiveness and feasibility of this method.

### 4.4. Simulation Verification

**Figure 19:**
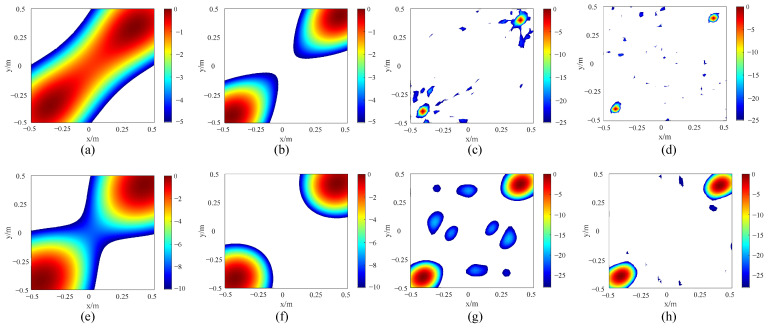
Simulation results of arrays’ recognition effect under two sound sources: (**a**) quasi improved LF array at 400 Hz, (**b**) improved LF array at 400 Hz, (**c**) quasi improved LF array at 7 kHz, (**d**) improved LF array at 7 kHz, (**e**) quasi improved HF array at 5 kHz, (**f**) improved HF array at 5 kHz, (**g**) quasi improved HF array at 20 kHz and (**h**) improved HF array at 20 kHz.

There are often multiple sound sources within the same field of view at the substation. At this time, it is necessary to accurately identify the number and location of sound sources. The anti-interference capability of the improved array is discussed by setting up two sound sources, which are symmetrically distributed. A simulation analysis is conducted on the sound source identification effect of the improved arrays at the cut-off frequencies. Figure 19 shows the simulation results.

As shown in Figure 19a,b, when the two contiguous sound sources are at 400 Hz, the side lobe suppression effect of the LF quasi improved array is not satisfactory, so a large part of the two acoustic field distribution cloud images overlap together, resulting in a misjudgment of the number and position of the sound sources. But the improved array can clearly distinguish the number of sound sources and locate the sound sources accurately by finding the deepest place of the red color. As shown in Figure 19c, when the sound sources are at 7 kHz, the true positioning spots of the quasi improved array are surrounded by false spots caused by the side lobes, which are again ghost images. As shown in Figure 19d, the spots of the improved array are smaller, and the ghost images are well suppressed. In addition, the improved HF array improvement effect is similar to the LF array.

### 4.5. Laboratory Verification

**Figure 20:**
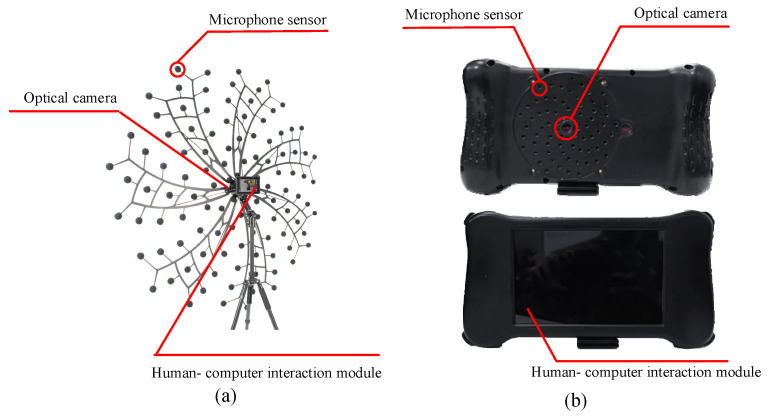
The appearance of the instruments: (**a**) LF instrument and (**b**) HF instrument.

Based on the above design method, LF and HF instruments are processed, respectively. The appearance of the instruments is shown in Figure 20. In the semi-anechoic room, using the processed instrument, the laboratory performance verification test is carried out for the steady and the transient sound sources.

#### 4.5.1. Steady Sound Source Test

**Figure 21:**
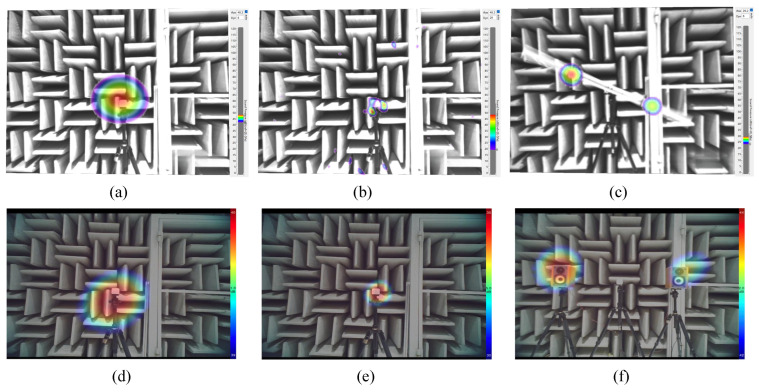
Test results of steady sound sources: (**a**) LF instrument Group 1, (**b**) LF instrument Group 2, (**c**) LF instrument Group 3, (**d**) HF instrument Group 1, (**e**) HF instrument Group 2 and (**f**) HF instrument Group 3.

Three test groups are designed, which are the single sound source (lower cut-off frequency *f*_min_), single sound source (upper cut-off frequency *f*_max_), and two sound source test group, as shown in Table 9. Group 1 and group 2 are designed to explore the positioning effect of the processed instrument under ideal conditions. In group 3, the weak sound source is designed to simulate the presence of the adjacent strong interference sound source. The two sound sources are broadband sound sources with equal energy density in the frequency domain of 400 Hz–20 kHz, which can ensure that the system is tested without biasing to a specific frequency. The test results are shown in Figure 21.

According to Figure 21, it can be seen from group 1 and group 2 that the spots are concentrated near the actual location in the acoustic–optical fusion image, and the color is the brightest at the actual location, indicating that both sets of instruments can accurately identify the single sound source at the cut-off frequency.

For group 3, there is no spatial confusion, and the spot color of the main sound source is brighter, indicating that the two sets of instruments can also clearly distinguish the main sound source and the adjacent interference sound source, which verifies that the instrument has a strong anti-interference ability and can meet the needs of the field test.

#### 4.5.2. Transient Sound Source Test

**Figure 22:**
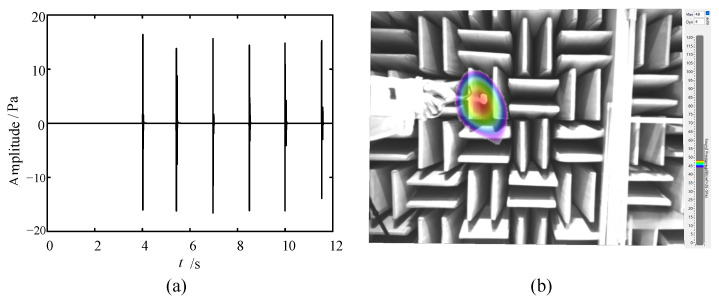
Test result of mechanical vibration: (**a**) time domain diagram and (**b**) acoustic-optical fusion image.

The transient sound source generated by typical intermittent mechanical vibration is simulated. The time domain diagram is shown in Figure 22a. The LF instrument is used; the test results are as shown in Figure 22b. It can be seen from Figure 22 that the instrument can successfully capture this mechanical vibration sound source and locate it accurately.

**Figure 23:**
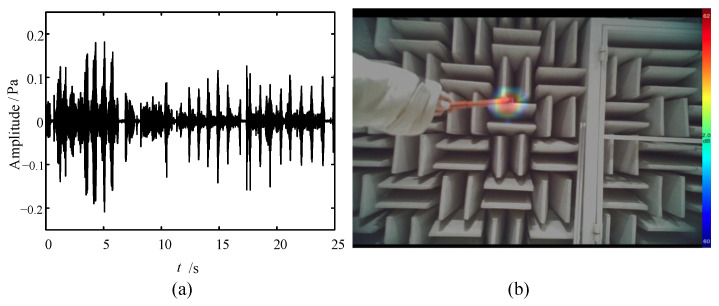
Test result of discharge: (**a**) time domain diagram and (**b**) acoustic-optical fusion image.

The transient sound source generated by typical discharge is simulated. The time domain diagram is shown in Figure 23a. The LF instrument is used; the test results are as shown in Figure 23b.

## 5. Conclusions

In this paper, the influence of design parameters such as the array shape, number of sensors, number of rings, and rotation angle of rings on array performance characteristic parameters is discussed. The optimization mathematical model is established, and an optimization design method of a microphone array suitable for complex sound sources in the substation is proposed. The optimal topology of the LF and HF array is obtained, and the following conclusions are drawn:

1. The array shape greatly affects both the −3 dB MLW and the MSL, and the circular array is the optimal shape.
2. The design parameters affect the imaging performance of the array to varying degrees, indicating that it is difficult to obtain the optimal array topology by an exhaustive method.
3. The two optimization algorithms including the DEA and the ACA are used to solve the same optimization problem, and the results are proved to be the global optimal solutions. Compared with the original array, the performance of the improved LF and HF array is promoted by 54% and 49%, respectively, which verifies the effectiveness and feasibility of the optimization method proposed in this paper.
4. Combined with the simulation analysis and laboratory test, a variety of defects in the field are simulated. It is verified that the improved array can not only accurately locate a steady or transient sound source but also accurately distinguish the main sound source from the interference of a contiguous sound source.

In the future, our research group will dig deeper into the research potential in this study. On the one hand, the influence of the sound source identification algorithm and acoustic signal refraction and reflection on imaging performance will be fully considered. On the other hand, more field abnormal sound samples will be collected to further assess the practical application effect of the improved array.

## Figures and Tables

**Table 1:** Summary of acoustic frequency domain characteristics.

Operating Condition	Dominant Frequency	POH/PEH	PAE
GIS			
Normal condition	100 Hz	POH is low	Concentrated below 2 kHz
Mechanical vibration	≥300 Hz	POH increases	Increase (300–2000 Hz)
Partial discharge	100 Hz	POH increases	Increase (5k–25k Hz)
Breakdown discharge	≥10 kHz	PEH decreases	Increase (≥5 kHz)
**Power transformer/reactor**			
Normal condition	100/200 Hz	POH is low	Concentrated below 2 kHz
Over excitation	≥300 Hz	POH increases	Increase (500–2000 Hz)
DC magnetic bias	≥300 Hz	POH increases	Increase (500–2000 Hz)
Iron-core multi-point earthing	Unchanged	Unchanged	Unchanged
Mechanical vibration	≥300 Hz	Unchanged	Increase (500–2000 Hz)
Partial discharge	Unchanged	POH increases slightly	Increase (≥5 kHz)
Breakdown discharge	≥10 kHz	POH increases	Increase (≥5 kHz)

**Table 2:** Comparison of arrays with different shapes’ evaluation indexes.

Array Shape	−3 dB MLW/m	MSL/dB
Crossed	0.1444	−5.7810
Rectangular	0.0822	−10.8860
Elliptic	0.1289	−15.8514
Circular	0.0778	−15.8520

**Table 3:** Optimization boundary conditions for LF instrument.

*m*_min_	*m*_max_	*n*_min_	*n*_max_	*r*_min_	*r*_max_	*θ*_min_	*θ*_max_
40	128	2	18	0.08	0.85	0	50

**Table 4:** Optimization results for LF instrument.

Serial Number of Rings	Radius of Rings	Number of Sensors	Rotation Angle
1	0.085	7	18.55
2	0.189	7	22.87
3	0.269	7	18.55
4	0.340	7	22.79
5	0.400	7	19.32
6	0.454	7	12.09
7	0.505	7	14.32
8	0.560	7	22.02
9	0.594	7	14.32
10	0.636	7	10.09
11	0.676	7	14.22
12	0.722	7	18.26
13	0.749	7	22.64
14	0.784	7	24.32
15	0.820	7	22.83
16	0.850	7	18.55

**Table 5:** MSL comparison of LF arrays.

Parameter	Original Array	Quasi-Improved Array	Improved Array	
			DEA	ACA
MSL	−17.95 dB	−25.93 dB	−27.67 dB	−27.67 dB
MSL promotion	/	44.46%	54.15%	

**Table 6:** Optimization boundary conditions for HF instrument.

*m*_min_	*m*_max_	*n*_min_	*n*_max_	*r*_min_	*r*_max_	*θ*_min_	*θ*_max_
40	128	2	18	0.01	0.068	0	50

**Table 7:** Optimization results for HF instrument.

Serial Number of Rings	Radius of Rings	Number of Sensors	Rotation Angle
1	0.015	9	13.06
2	0.026	9	16.24
3	0.036	9	22.23
4	0.043	9	24.09
5	0.046	9	23.02
6	0.054	9	22.14
7	0.059	9	18.68
8	0.068	9	23.87

**Table 8:** MSL comparison of HF arrays.

Parameter	Original Array	Quasi-Improved Array	Improved Array	
			DEA	ACA
MSL	−17.97 dB	−25.65 dB	−26.77 dB	−26.77 dB
MSL promotion	/	42.74%	48.97%	

**Table 9:** Test groups designed for steady sound sources.

Group		Group 1	Group 2	Group 3
Number of sound sources		1, at *f*_min_	1, at *f*_max_	2
LF instrument	Frequency	400 Hz	7 kHz	400 Hz–20 kHz
	Sound pressure ratio	/	/	1:0.8
HF instrument	Frequency	5 kHz	20 kHz	400 Hz–20 kHz
	Sound pressure ratio	/	/	1:0.8

## Data Availability

Data are contained within the article.
